# Sarcomatoid carcinoma of the renal pelvis: Experience of multiple cases over a ten-year period

**DOI:** 10.3892/ol.2013.1396

**Published:** 2013-06-13

**Authors:** SHANWEN CHEN, GEMING CHEN, DAN XIA, JUN LI, SHUO WANG, BAIHUA SHEN, BAIYE JIN

**Affiliations:** 1 Departments of Urology, Medical School of Zhejiang University, Hangzhou, Zhejiang 310031, P.R. China; 2 Pathology, The First Affiliated Hospital, Medical School of Zhejiang University, Hangzhou, Zhejiang 310031, P.R. China

**Keywords:** renal pelvis, sarcomatoid carcinoma, nephroureterectomy, prognosis

## Abstract

Sarcomatoid carcinoma of the renal pelvis is a rare clinical entity. To the best of our knowledge, only 14 cases of this type of neoplasm have been reported in the literature to date. In the present study, the records at The First Affiliated Hospital, Medicine School of Zhejiang University (Hangzhou, Zhejiang, China) between 2000 and 2010 were reviewed to identify patients with primary renal pelvis sarcomatoid carcinoma (RPSC). A particular emphasis was placed on the treatment, recurrence and survival outcome. Eight patients with RPSC were identified and treated with nephrectomy or nephroureterectomy. All of the patients presented with Grade 3 RPSC. According to the TNM classification system, 2 patients were in stage pT2, 5 in stage pT3 and 1 in stage pT4. Adjuvant chemotherapy was administered to four patients, and the mean follow-up period was 27.5±41.0 months. In total, 6 patients succumbed to the disease with a mean survival time of 7.7±5.3 months (range, 1–18 months), while 2 patients were free of disease at 54 and 120 months, respectively, following treatment. The mean disease-specific survival time was 27.5±41.0 months and the 1-year recurrence-free survival, 1-year survival and overall survival rates were 37.5, 37.5 and 25%, respectively. The present analysis suggests a poor prognosis for the majority of RPSC patients, most likely resulting from the advanced stage of the disease at diagnosis and a poor response to systemic therapy. To improve the survival rate of RPSC, it is therefore essential to perform an early diagnosis and early radical surgery. Intravesical instillation is not essential following surgery.

## Introduction

Transitional cell carcinoma is the most common histological type of urinary tract cancer. Squamous cell carcinoma and adenocarcinoma form the second and third most common histological types, respectively. Renal pelvis sarcomatoid carcinoma (RPSC) is an extremely rare variant of urinary tract cancer, which possesses a high-grade malignant neoplasm and a poor prognosis. To the best of our knowledge, only 14 cases of RPSC have been reported in the literature to date, usually as case reports or small series (1–9). In the present study, we report our experience with 8 cases of RPSC. A particular emphasis was placed on the treatment, recurrence and survival outcome of RPSC.

## Patients and methods

### Patients and methods

Between January 2000 and December 2010, 8 males with RPSC (age range, 38–78 years; mean age, 65.3±14.0 years) were registered in the surgical pathology files at The First Affiliated Hospital, Medicine School of Zhejiang University (Hangzhou, Zhejiang, China), including 1 previously reported case (10). Formalin-fixed paraffin-embedded tissue sections stained with hematoxylin and eosin were available for examination for each case. The sarcomatoid carcinoma in each instance consisted of a spindle cell malignancy with or without coexistent overt urothelial carcinoma. Light microscopical or immunohistochemical evidence of epithelial differentiation in the spindle cells was also observed. The patient records were reviewed to assess the tumor type, treatment modalities, recurrence and survival parameters. Tumor recurrence was detected by routine follow-up imaging performed at 3–6-month intervals. The pathological features were retrospectively evaluated and confirmed by a urological pathologist. The institutional review board of the The First Affiliated Hospital, Medicine School of Zhejiang University approved the retrospective study. Written informed consent was obtained from the patient or patient’s family.

## Results

### Diagnosis

Of the 342 evaluated patients that were treated for tumors of the renal pelvis, 8 (2.3%) were diagnosed with primary RPSC. The tumor characteristics and patient outcomes are shown in Table I. Among the 8 patients diagnosed with sarcomatoid carcinoma, the male-to-female ratio was 8:0. The symptoms observed in the 8 patients included hematuria in 7 cases and abdominal pain in 1 case. A tumor of the renal pelvis was identified in all patients by contrast-enhanced computed tomography. Malignancy was diagnosed cytologically with renal pelvis lavage in 2 cases (including 1 case with sarcomatoid carcinoma) prior to surgery. Immunohistochemical studies confirmed the diagnosis of sarcomatoid carcinoma. Prior to surgery, 3 patients were in stage pT2 and 5 were in stage pT3, according to the TNM classification system.

Of the 8 patients diagnosed with RPSC, 3 patients were treated with nephrectomy (2 patients with nephrectomy plus chemotherapy) and 5 patients with nephroureterectomy (2 patients with nephroureterectomy plus chemotherapy). The gross appearance of the sarcomatoid carcinoma was described in 8 cases. The mean tumor size was 5.8±4.5 cm (range, 1.5–16.0 cm) at the greatest dimension. In total, 5 lesions were polypoid and 3 were nodular. A pure spindle cell carcinoma without any apparent epithelial components was noted in 1 case, spindle and transitional cell carcinoma in 4 cases and spindle and squamous cell carcinoma in 3 cases. According to the TNM classification system, 2 patients were now diagnosed with stage pT2, 5 with stage pT3 and 1 with stage pT4.

### Survival

The mean follow-up time was 27.5±41.0 months. A total of 6 patients succumbed to the disease with a mean survival time of 7.7±5.3 months (range, 1–18 months; Table I), while 2 patients (cases 4 and 7) were free of disease at 54 and 120 months, respectively. The mean disease-specific survival time was 27.5±41.0 months. The 1-year recurrence-free survival, 1-year survival and overall survival rates were 37.5, 37.5 and 25%, respectively. A comparison between the patients who underwent surgery alone and those who underwent surgery plus chemotherapy demonstrated no evident difference in survival (Table II).

## Discussion

Sarcomatoid carcinoma has been reported in a variety of organs, including the urinary bladder (11,12), kidney (13) and female genital tract (14). RPSC is an extremely rare entity, with only 14 confirmed cases reported in the literature since 1961 (1–9).

The characteristics of the 22 reported patients (including the patients examined in the present study) with RPSC are shown in Table I and Table III. RPSC was more common in males than females (4:1 ratio in previously reported studies, 8:0 ratio in the present study) and more common in the right side compared with the left side (5:2 ratio in previously reported studies, 5:3 ratio in the present study). The most common symptom of RPSC was gross hematuria.

The prognosis for patients diagnosed with RPSC is poor. Several factors contribute to this poor prognosis, including diagnosis at an advanced stage of the disease, high-grade features, a large tumor and a short progression time despite additional therapy. The previously reported 13 cases of RPSC were of metastatic disease or advanced tumor involvement of the renal parenchyma, which had an extremely poor prognosis (1–8). Among these 13 patients, 8 succumbed to disease within 2 years of diagnosis (range, 2–20 months) and adjuvant chemotherapy provided no response or survival advantage when used (7). Thiel *et al* reported the 14th case of urothelial-confined and organ-confined RPSC, which presented without recurrence for >16 months (9). A nephrectomy or nephroureterectomy was performed in all of the cases of RPSC examined in the present study; however, 6 patients (5 in TNM stage pT3 and 1 in TNM stage pT4) succumbed to the disease within 18 months (range, 1–18 months; mean, 7.7 months). The patients in the present study (TNM stage pT3 or pT4 disease) rarely had a prolonged survival time despite undergoing surgery. Of the 2 patients (TNM stage pT2) who were alive without recurrence at the final follow-up, 1 did not receive chemotherapy. Adjuvant chemotherapy exerted a minimal effect in the present study. Due to the limited number of patients and the nature of a retrospective analysis, a conclusion should not be made with regard to the role of adjuvant chemotherapy, as the patients treated this way may have had a more advanced stage of disease resulting in a selection bias. In addition, no patient in this study developed recurrence in the bladder, supporting the hypothesis that non-urothelial carcinoma has a different tumor biology compared with urothelial carcinoma. Therefore, intravesical instillation is not essential following surgery.

As RPSC is known for its aggressive nature, insensitivity to adjuvant therapy and adverse outcomes, it is essential to conduct an early diagnosis and early radical surgery to improve the survival outcome (7,8). In the present study, the 2 cases of urothelial-confined and organ-confined RPSC (cases 4 and 7) did not recur for >4 years following radical surgery. Efforts aimed at improving the accuracy of diagnosis at presentation and the consequent treatment strategies are required. The main obstacle for the accurate diagnosis and treatment of RPSC is that the presentation of non-urothelial tumors is usually similar to that of urothelial tumors. Any findings that suggest the possibility of RPSC should subsequently invoke a more thorough investigation implementing a repeat biopsy with specific immunohistochemical stains. These findings may include the following: abnormal cytology results that are incongruent with tumors of the renal pelvis (as in the present study, immunocytochemistry with markers of sarcomatoid carcinoma aids discrimination); atypical radiological characteristics or any evidence of metastatic disease despite the radiological appearance of small-volume disease. Following a clinical presentation that suggests RPSC, neoadjuvant strategies may aid in the identification of those patients who are the best candidates for surgery and also aid in avoiding unnecessary surgery in patients where it is likely that their disease will progress rapidly. Patients with TNM stage pT2 should be treated with nephroureterectomy, patients with TNM stage pT3 should be treated with nephrectomy and patients with TNM stage T4 should avoid surgery. However, this hypothesis remains to be verified.

In conclusion, RPSC is rare. The present study analysis suggests a poor prognosis for the majority of patients with RPSC, most likely as a result of the advanced stage of the disease at diagnosis and the poor response to systemic therapy. It is essential to conduct an early diagnosis and early radical surgery to improve the survival outcome. Intravesical instillation is not essential following surgery.

## Figures and Tables

**Table I.** Patient characteristics of the cases of RPSC reported in the present study.

No.-age-gender-side	Symptoms	Date of surgery	T/Fu/Mo	Tumor size (cm)	Histology	Stage	Subsequent treatment	Recurrence (time after surgery, months)
1-77-M-L	Abdominal pain	Oct 2010	Nu/STD/8	16.0×12.0×9.0	Spindle/transitional	pT3G3N0M0	No	7
2-78-M-L	GH	Dec 2009	Nu/STD/3	6.0×3.5×3.0	Spindle/squamous	pT4G3N0M1	No	1
3-77-M-R	GH	Aug 2007	N/STD/18	3.5×3.1×2.0	Spindle/squamous	pT3G3N0M0	No	17
4-71-M-R	GH	Nov 2006	Nu/NED/54	1.5×1.5×1.2	Spindle/transitional	pT2G3N0M0	No	No
5-53-M-R	GH	Mar 2006	N/STD/6	6.8×3.6×2.1	Spindle	pT3G3N0M0	Yes	3
6-62-M-R	GH	Jul 2004	N/STD/6	5.9×3.6×3.1	Spindle/squamous	pT3G3N1M0	Yes	1
7-38-M-R	GH	May 2001	Nu/NED/120	2.2×1.8×1.6	Spindle/transitional	pT2G3N0M0	Yes	No
8-66-M-L	GH	Apr 2000	Nu/STD/5	4.2×2.8×1.5	Spindle/transitional	pT3G3N1M0	Yes	3

No., number (patient); T/Fu/Mo, treatment/follow-up/months; M, male; L, left; R, right; GH, gross hematoma; N, nephrectomy; Nu, nephroureterectomy; STD, succumbed to disease; NED, no evidence of disease; RPSC, renal pelvis sarcomatoid carcinoma.

**Table II.** Patient outcomes of the cases of RPSC reported in the present study.

Variable	All	Surgery alone	Surgery plus chemotherapy
Total patients, n	8	4	4
Alive, n	2	1	1
NED, n	2	1	1
Follow-up, months^a^	27.5	20.8	34.3
Follow-up of patients who remained alive, months^a^	87.0	54.0	120.0
Follow-up of patients who succumbed to disease, months^a^	7.7	9.7	5.7
Survival, months^a^	27.5	20.8	34.3
1-year survival rate, %	37.5	50	25
RFS, months^a^	25.8	19.8	31.8
1-year RFS rate, %	37.5	50	25
DSS, months^a^	27.5	20.8	34.3
1-year DSS rate, %	37.5	50	25

a Numbers represent mean values, unless otherwise specified. NED, no evidence of disease; RFS, recurrence-free survival; DSS, disease-specific survival; RPSC, renal pelvis sarcomatoid carcinoma.

**Table III.** Patient characteristics of previously reported cases of RPSC.

Patient no.	First author/s (ref.)	Age/gender/side	Histology	T/Fu/Mo
1	Fauci *et al* (1)	61/F/R	TCC/pleomorphic	Nu/NED/6
2	Tarry *et al* (2)	61/F/R	TCC/giant-cell tumor-like	Nu/NED/20
3	Piscioli *et al* (3)	62/M/R	TCC/rabdomyosarcomatous tumor-like	Nu/NR/NR
4	Wick *et al* (4)	45/M/L	TCC/anaplastic-spindle	Nu/STD/22
5	Wick *et al* (4)	65/F/R	SQ/spindle	Nu/STD/7
6	Tajima and Aizawa (5)	66/M/R	TCC/adenocarcinoma/anaplastic-spindle	Nu/NED/12
7	Suster and Robinson (6)	85/M/R	Spindle	Nu/NED/3
8	Lopez-Beltran *et al* (7)	65/M/L	Spindle/anaplastic	N/STD/20
9	Lopez-Beltran *et al* (7)	66/M/R	Spindle/anaplastic	N/STD/6
10	Lopez-Beltran *et al* (7)	66/F/R	Spindle/anaplastic	N/STD/18
11	Lopez-Beltran *et al* (7)	82/F/R	Spindle/myxoid	Nu/STD/6
12	Lopez-Beltran *et al* (7)	79/M/R	Spindle/myxoid	N/STD/6
13	Hisataki *et al* (8)	43/F/L	TCC/spindle	N/STD/14
14	Thiel *et al* (9)	65/M/L	Spindle/osteoclast-type giant cells	Nu/NED/16

F, female; M, male; R, right; L, left; TCC, transitional cell carcinoma; SQ, squamous differentiation; T/Fu/Mo, treatment/follow-up/months; N, nephrectomy; Nu, nephroureterectomy; NED, no evidence of disease; NR, not reported; STD, succumbed to disease; RPSC, renal pelvis sarcomatoid carcinoma
